# Processed Meats as Delivery Systems for Nutraceuticals: Technological Advances, Bioavailability, Health Benefits, and Future Perspectives

**DOI:** 10.3390/foods15183330

**Published:** 2026-09-20

**Authors:** Tulay Elal Mus

**Affiliations:** Department of Food Processing, Vocational School of Karacabey, University of Bursa Uludag, 16300 Bursa, Türkiye; tulayelalmus@uludag.edu.tr; Tel.: +90-224-294-2662

**Keywords:** meat products, bioactive ingredients, functional foods, nanoencapsulation, food matrix

## Abstract

Processed meat products have traditionally been developed to improve shelf life, microbial safety, and sensory quality; however, recent progress in food technology has expanded their role as potential delivery systems for nutraceuticals. The unique physicochemical characteristics of meat product matrices, including protein gel structure, lipid emulsions, and hydration capacity, offer an effective environment for protecting bioactive compounds during processing, storage, and gastrointestinal digestion. This review article has provided an overview of the major nutraceuticals in meat products. The technofunctional effects of dietary fibers, polyphenols, plant-based oils, probiotics, postbiotics, herbs, and spices, including improvements in oxidative stability, microbial safety, lipid profile, texture, sensory quality, and shelf life, are critically discussed together with their interactions within meat matrices. Novel delivery technologies, nutraceutical stability in meat matrices, and bioavailability of compounds are highlighted. Current evidence indicates that nutraceuticals substantially preserve bioactivity in meat matrices while improving their bioaccessibility. Clinical reports suggest potential effects on health; however, the scarcity of studies is insufficient to establish consistent or long-term benefits. Rapid progress is being made in the design of meat formulations, but further work on advanced delivery technologies, human intervention studies, and consumer-oriented products is still needed. Consequently, processed meats provide a convenient and promising multifunctional matrix for delivering nutraceuticals.

## 1. Introduction

Over the last two decades, consumer demand has shifted significantly from foods intended to satisfy hunger to products that promote health, prevent chronic disorders, and enhance the quality of life [1]. Increasing consciousness of the relationship between diet and non-contagious diseases, together with an aging population and rising healthcare costs, has stimulated interest in foods enriched with bioactive compounds that deliver physiological benefits beyond basic nutrition [2]. This trend has accelerated the development of functional foods incorporated with nutraceuticals, which are among the fastest-growing sectors of the worldwide food industry. Nutraceuticals are a hybrid of nutritional components and pharmaceutical agents that are biologically active and capable of maintaining health conditions and providing benefits [3]. Despite the increasing availability of nutraceutical compounds, their successful incorporation into foods remains a remarkable technological challenge because many bioactive ingredients exhibit poor stability, have low bioavailability, affect sensory attributes, or interact with food components [4]. Consequently, considerable attention has been directed toward revealing suitable food matrices that protect nutraceuticals during processing, storage, and gastrointestinal digestion while ensuring consumer acceptance [5].

**Figure 1:**
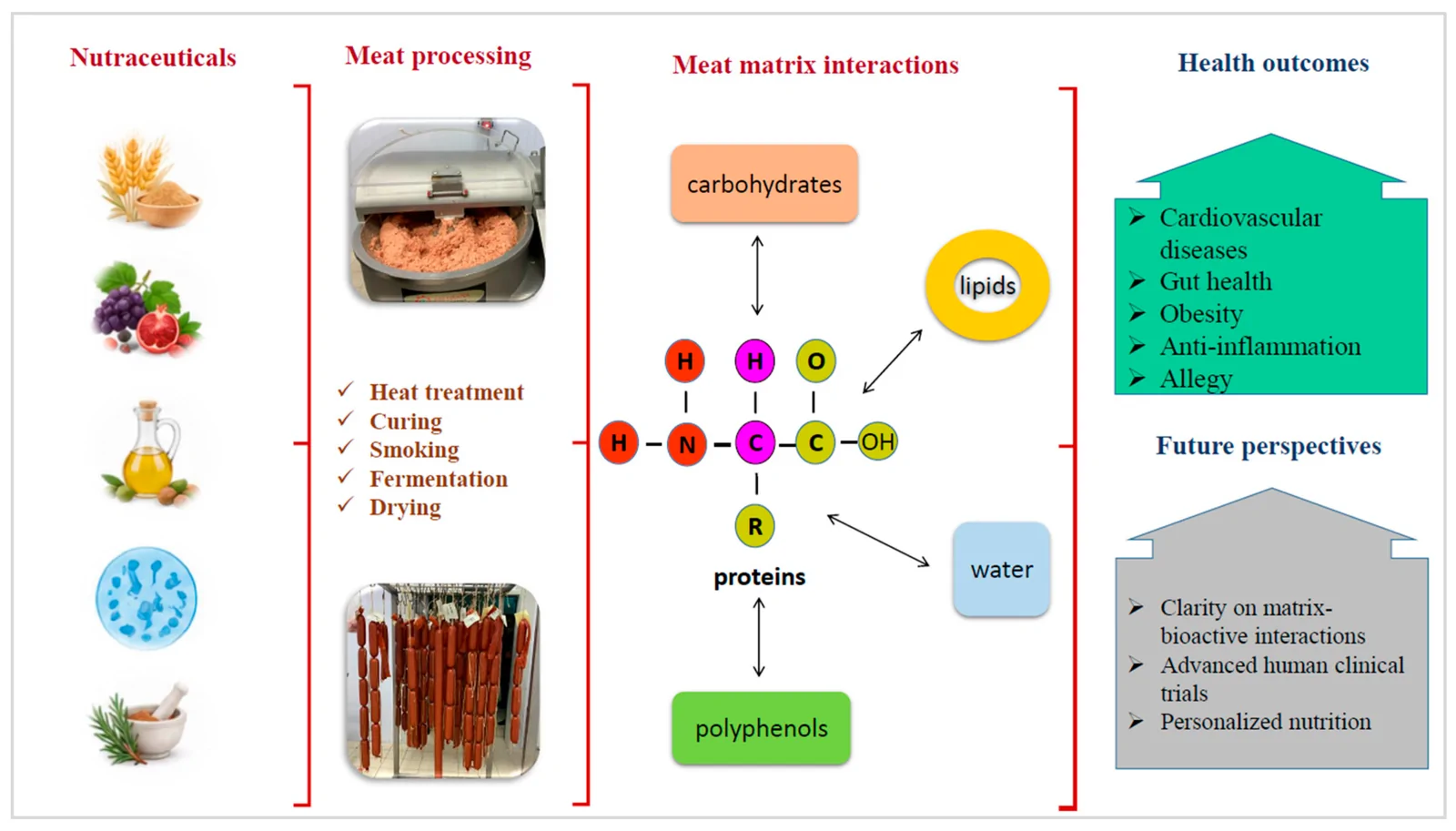
Schematic representation of nutraceutical delivery through the processed meat matrix.

Meat is a primary source of high-quality dietary protein, amino acids, vitamins, minerals, and essential nutrients, as well as a reservoir of bioactive peptides that support human physiology [6]. In the meat matrix, proteins serve as vectors for flavor compound interactions, contributing to flavor retention and stability, while lipids co-occur with proteins during heat treatment and bind volatile compounds [7]. Furthermore, proteins have gelation, emulsification, water-holding, oil-binding, and foaming technofunctional properties that drive the success of food formulations [8]. These properties make meat products a suitable matrix for the delivery of nutraceuticals. Otherwise, meat is susceptible to oxidative reactions and microbial growth due to its rich nutritional content and high moisture, which can lead to a deterioration in taste and a reduction in nutritional quality, thereby diminishing shelf life. To protect meat properties and extend shelf life, meat is processed with chemical additives such as sodium nitrite and nitrate, as well as synthetic antioxidants, which are widely used in the production [9]. Epidemiological studies have associated higher consumption of processed meat with an increased risk of chronic diseases, particularly colorectal, breast, and lung cancers, cardiovascular diseases, and type 2 diabetes. However, these associations may not be interpreted as indicating an identical risk among all processed meat products, as their composition, heat-treatment conditions, and degree of processing vary considerably. Mechanisms that pose a risk include high sodium and saturated fat contents in some formulations, exposure to nitrite- and nitrate-derived compounds, lipid and protein oxidation products, and other processing-derived molecules [10]. Nevertheless, epidemiological, mechanistic, and causal evidence differ in strength and should be considered accordingly. In this context, reformulation strategies incorporating bioactive and functional ingredients have increasingly been studied to improve the nutritional profile and technofunctional properties of processed meat products [11,12,13,14,15]. These numerous studies have focused on the reformulation of specific meat products using particular classes of bioactive compounds. Additionally, previous reviews [16,17,18,19,20] have addressed functional meat reformulation and individual classes of bioactive ingredients for improving the nutritional quality and safety of processed meat products. However, comparatively limited attention has been given to the integrated role of the processed meat matrix as a delivery platform for nutraceuticals, particularly regarding the relationships among matrix composition, technological performance, processing stability, gastrointestinal bioaccessibility and bioavailability, and evidence of potential health benefits. A critical synthesis connecting these dimensions is therefore needed to clarify both the opportunities and the limitations of nutraceutical-enriched processed meat products. Accordingly, this review aims to (i) characterize the physicochemical features of processed meat matrices relevant to nutraceutical incorporation and delivery; (ii) critically evaluate the technological benefits associated with major classes of incorporated nutraceuticals and functional ingredients; (iii) examine the stability, bioaccessibility, and bioavailability of these compounds during processing, storage, and gastrointestinal digestion; and (iv) assess the current literature for potential health effects while identifying key technological, clinical, and research gaps for future development. Figure 1 shows a schematic representation of this review.

### Literature Search Strategy

The Google Scholar, PubMed, Web of Science, and Scopus databases were used to retrieve documents published between 2016 and 2026. The search keywords were “nutraceuticals” or “meat products” or “processed meat”. Additionally, combinations of keywords including dietary fiber, polyphenols, fatty acids, probiotics, postbiotics, muscle foods, meat by-products, salami, sausage, sucuk, nugget, hamburger, bioavailability, health impacts, food applications, and bioaccessibility were also used to expand the search for more papers. The retrieved articles were eliminated for their relevance to the issue, and only then were they cited in this review.

## 2. Processed Meat Products as Functional Delivery Platforms

Processed meats were originally developed primarily to extend the shelf life of raw meat, ensure microbial safety, and enhance sensory attributes through salting, drying, smoking, fermentation, and curing [21]. These techniques have been successful in controlling microbial growth, which can adversely affect the sensory and nutritional quality of meat. Today, the use of emerging techniques, such as pulsed electric fields, high-pressure processing, intelligent cutting, ultrasonication, and digital technologies in meat processing, offers novel approaches that can improve microbiological safety while better preserving meat quality [22]. The advantages of emerging methods compared to traditional processing are (i) better preservation of sensory and nutritional properties, (ii) efficient microbial decontamination while minimizing thermal treatment, and (iii) potential for minimally processed and ready-to-eat products [23]. Furthermore, novel trends such as clean-label principles, personalized nutrition, inclusion of functional ingredients in formulations, and interest in sustainability are major frontiers driving the food industry [24]. In this context, healthier foods (lower in fat, sugar, and salt, etc.) reduce the risk of various chronic diseases [25], while functional foods have been innovated to offer specific health benefits beyond basic nutrition [26].

A food matrix is a structural organization of food components at the microstructural and macromolecular levels that modulate their physicochemical properties, sensory attributes, and nutritional functionality. Processed meat products constitute protein and an emulsified fat matrix, and their nutritional implications include improved absorption of nonheme iron and improved protection for probiotics [27]. The quality and performance of processed meats depend on the properties of myofibrillar, sarcoplasmic, and connective tissue proteins, which determine attributes such as texture, water-holding capacity, and structural integrity [28]. The functional characteristics of meat products predominantly depend on salt-soluble myofibrillar proteins that form and strengthen heat-triggered three-dimensional networks during heat treatment and following chilling [29]. Protein–carbohydrate interactions contribute to water retention, textural properties, shelf stability, and product quality in processed meats. Particularly, protein–starch interactions lead to gelation, retrogradation, and thickening, while interactions between proteins and pectin/cellulose improve texture and moisture retention in meat products [30]. Proteins and polyphenols interact through covalent and non-covalent bonds. Protein/phenolic ratio, pH, polyphenol properties, and binding affinity drive the strength of conjugates’ antioxidant activity [8]. In a processed meat matrix, polyphenols affect protein oxidation in different ways. For example, rutin improves the structural stability of sarcoplasmic and myofibrillar proteins, and quercetin inhibits the formation of dimeric tyrosine during myofibrillar oxidation. At the same time, caffeic acid delays the accumulation of Schiff bases in place of carbonyls [31]. Protein–lipid interactions during processing enhance gel properties, improving color, water-holding capacity, and sensory attributes in meat products [32]. Myofibrillar proteins naturally bind to diacylglycerols, and their ability to form gels is crucial for meat processing, affecting the final meat product’s sensory and textural attributes. Furthermore, immobilized fat droplets within the protein matrix are critical for decreasing cooking loss, thereby improving water retention, water-holding capacity and product palatability. The interaction between diacylglycerols and myofibrillar proteins leads to the formation of stable gels, affecting the texture, water-holding capacity, and quality of meat products [33]. Most processed meat products are oil-in-water emulsions, and structured oils have the potential to enable the development of innovative food products [34]. Therefore, processed meat products possess a unique, highly organized food matrix that provides structural and physicochemical advantages for incorporating, protecting, and delivering nutraceutical compounds. The bioactive nutraceutical ingredients are highly susceptible to degradation by various factors, including enzymes, thermal treatment, pH, oxidation, light, and/or hydrolysis [35]. The complex meat matrix can provide a protective microenvironment that reduces exposure to these detrimental factors. However, interactions between nutraceuticals and the meat matrix should not be regarded as uniformly beneficial. Protein–polyphenol interactions may protect phenolic compounds against oxidation or processing-induced degradation, but strong covalent or non-covalent binding may also modify their solubility, digestibility, and enzymatic accessibility. The substantiality and tendency of these effects depend on the molecular structure and concentration of the polyphenol, protein characteristics, pH, ionic strength, processing conditions, and the types of interactions formed [36]. Similarly, the lipid phase of meat products can facilitate the incorporation and dispersion of lipophilic bioactive compounds and provide an appropriate environment for the delivery of lipid-soluble bioactives [37]. Nevertheless, increasing the proportion of unsaturated lipids may increase susceptibility to lipid oxidation and the formation of undesirable oxidation products during processing and storage.

Protein aggregation is a biological process that, depending on temperature, heating time, protein composition, pH, ionic strength, and redox conditions, may involve hydrophobic interactions, hydrogen bonding, electrostatic interactions, and the formation or rearrangement of disulfide bonds. Three-dimensional gel networks aggregate proteins, thereby determining the physical organization of processed meat products and serving as delivery vehicles for pharmaceuticals and nutraceuticals [38]. From a nutraceutical-delivery perspective, heat-induced protein aggregation may have contrasting consequences. Formation of a protein network may physically entrap incorporated biocompounds and potentially protect susceptible compounds against migration or degradation during processing and storage. Conversely, extensive aggregation or strong interactions between bioactives and denatured proteins may restrict their subsequent release during gastrointestinal digestion. Thermal aggregation may also modify protein accessibility to digestive enzymes, thereby altering matrix fracture and the release kinetics of associated compounds. Thus, raised retention of a nutraceutical within heat-treated processed meat should not necessarily be interpreted as increased gastrointestinal bioaccessibility. The net effect depends on the type of bioactive compound, the nature and extent of protein aggregation, and the processing and digestive conditions.

## 3. Nutraceuticals and Functional Ingredients Incorporated into Processed Meats

For this review, nutraceuticals are considered food-derived bioactive compounds or preparations intended to provide physiological benefits beyond their basic nutritional value. This concept should be distinguished from functional ingredients, which are incorporated into food formulations to improve nutritional composition or provide specific functional properties, and from technological ingredients, whose primary purpose is to modify processing performance, structure, texture, stability, or yield. These categories are not necessarily mutually exclusive, as a single ingredient may exert nutritional, physiological, and technological effects simultaneously. Accordingly, the classification used in this review is based primarily on the intended role and reported outcomes of each ingredient in the original studies. Dietary fibers, polyphenols, probiotics, postbiotics, herbs, and spices may provide bioactive or physiological functions in addition to their technological effects, whereas structured lipid systems such as oleogels, gelled emulsions, and bigels are more appropriately considered reformulation or delivery systems rather than nutraceuticals per se. Plant-based oils may similarly serve as both sources of bioactive lipids and reformulation ingredients to modify the fatty acid profile of processed meat products. The following subsections, therefore, consider both the bioactive potential of incorporated ingredients and their technological roles within the meat matrix. The reported effects of incorporated ingredients are considered across the technological, preservation, and nutritional/functional categories throughout this review.

### 3.1. Dietary Fibers

Dietary fiber is defined as plant-derived carbohydrate oligomers and polymers that are non-digestible and absorbable in the small intestine and are subjected to partial or complete fermentation in the colon [39]. Dietary fibers can be divided into low- and high-molecular-weight categories based on chain length and degree of polymerization. Fibers with low molecular weight exhibit better solubility and a high degree of fermentation [40]. Also, the particle size and integrity of dietary fibers impact the dissolution of soluble fibers and their fermentation. Small particles have been associated with increased concentrations of short-chain fatty acids (SCFAs) and greater bacterial fermentation [41]. Structural characteristics such as crystallinity, porosity, degree of branching, and poor wettability of fibers substantially influence their interactions with intestinal microorganisms [42]. The aim of adding dietary fibers to meat products is to enhance their physicochemical characteristics, chemical composition, textural features, and sensory attributes, as well as to act as an effective extender/binder/filler to decrease the production cost by lowering the cooking loss, as well as by reducing the lean meat, and also as a fat replacer to alleviate animal fat in the innovative processed meats [43]. Additionally, the use of fiber-rich fruit and vegetable by-products in meat processing enhances meat products’ quality and functional characteristics, while supporting environmental sustainability by reducing waste [44]. Table 1 [12,13,45,46,47,48,49,50,51,52,53,54,55,56,57,58] summarizes research progress of dietary fiber applications in processed meat products, along with recent references. The incorporation of cereal, fruit, vegetable, coffee, and nut skin-derived fibers into meat products confers many functional benefits, such as decreased lipid oxidation, improved water retention, lower oil absorption, enhanced texture, and sensory characteristics.

### 3.2. Polyphenols

Polyphenols are essential plant-based phenolic compounds that include aromatic rings and hydroxyl groups in their structure. Flavonoids, phenolic acids, polyphenolic amides, tannins, curcuminoids, lignans, and stilbenes are types of polyphenols [59]. These phytochemicals are abundant in dietary sources and exhibit many pharmacological effects, particularly antimicrobial and antioxidant activities [60]. On the other hand, polyphenols exhibit pro-oxidant activity under certain conditions, particularly high pH, high phenolic concentrations, and the presence of redox-active transition metals such as iron and copper. This activity is based on the generation of a phenoxyl radical or a redox complex with a transition-metal ion, thereby allowing phenoxyl radicals to react with oxygen [61,62]. These mechanisms help maintain cured color, reduce lipid oxidation, and mitigate N-nitrosamine formation in cured meats, making them natural alternatives to synthetic additives for preservation in clean-label foods [63]. Moreover, dietary flavonoids and their metabolites contribute to regulating the gut microbiota of consumers [64,65]. They are incorporated into processed meats to prevent or delay lipid oxidation, inhibit microbes, extend shelf life, and preserve product quality [66]. However, these phytochemicals, as well as their derivatives, degrade in food depending on the processing method used during production. Non-thermal food processing technologies, such as pulsed electric fields, ultrasound, and radiation processing, maintain or increase polyphenol content, whereas the fermentation and heat-treatment processes reduce the product’s polyphenol content [67]. Numerous studies have investigated the incorporation of polyphenol-rich foods and their by-products into processed meat formulations, which are presented in Table 2 [68,69,70,71,72,73,74,75,76,77,78]. Polyphenol incorporation primarily improved lipid oxidation characteristics and contributed to many technofunctional properties of the meat products. Additionally, the presence of polyphenols enhances gastrointestinal digestion and affects myofibrillar protein structure, making the meat matrix a convenient environment for the delivery of polyphenols.

### 3.3. Plant-Based Oils and Structured Lipid Systems

Animal fats, due to their high saturated fatty acid (SFA) content, have been replaced with plant-based oils rich in PUFAs and MUFAs in processed meat products to improve their lipid profiles and lipid health indices [79]. Fat is a major ingredient in the development of the palatability, flavor, texture, and juiciness of meat products. Plant-based oils are structured in the meat matrix as liquid, encapsulated, gelled emulsions, pre-emulsified, or oleogel forms to develop functional foods [80]. As shown in Table 3 [14,15,52,79,81,82,83,84,85,86,87], the incorporation of plant-based oils, oleogels, gelled emulsions, and bigels into meat products leads to higher levels of PUFAs and MUFAs and enhanced lipid indices compared with the control formulation, offering potential health benefits. Several technological challenges exist in plant-based oil applications in food matrices. Unsaturated fatty acids are prone to lipid oxidation, the primary factor responsible for oil degradation and the generation of off-flavor compounds from secondary oxidation products [88]. Therefore, the inclusion of PUFA-rich plant-based oils should not be interpreted as an unequivocal improvement in overall product quality. Consequently, they may generate a nutritional–technological trade-off.

### 3.4. Probiotics and Postbiotics

Probiotics are live microorganisms that benefit host health, and their inactivated (intact and nonviable) forms are classified as paraprobiotics, whereas metabolic products or by-products secreted by probiotics or released after bacterial lysis are categorized as postbiotics [89]. Paraprobiotics and postbiotics, derivatives of probiotics, are emerging functional ingredients owing to their ability to modulate the digestive system and promote host health. They do not require strict production and storage conditions, unlike probiotics [90]. Maintaining probiotic viability during processing, storage, and digestion remains a major challenge, but meat products, particularly fermented meats, are a suitable delivery system for probiotics without adverse effects [91]. Unlike probiotics, postbiotics have high safety, better processability, and stability across a wide range of temperatures and pH levels, and most of them have negligible interactions with other components of the food matrix [92]. Moreover, bacteriocins are probiotic-origin antimicrobial peptides that provide food safety by inhibiting spoilage and pathogenic microorganisms. These compounds secreted by probiotics are crucial for modulating the host’s native microbiota, thereby ameliorating dysbiosis and enhancing overall intestinal and host health [93]. Although only nisin and pediocin PA-1 among the isolated bacteriocins have been approved by the FDA for use in the food industry, nisin interacts with and becomes entrapped by the lipids in meat products, thereby reducing its effectiveness in these products [92]. Table 4 [94,95,96,97,98,99,100,101,102,103,104,105] presents results from recent studies on the effects of probiotics and postbiotics on the functionality of processed meat products. The variety of technofunctional effects on meat products depends on the probiotic strain, production process, pH, and other ingredients.

### 3.5. Herbs and Spices

Herbal nutraceuticals are foods prepared from plants and/or their roots, seeds, bark, fruits, leaves, or flowers [106], and essential oils derived from these parts of plants [107]. Herbs and spices contain varying levels of polyphenols, flavonoids, and terpenoids that make important contributions to culinary applications [108]. Spices are widely incorporated into processed meats not only to enhance flavor, color, and nutritional value but also to provide antioxidant, antimicrobial, and pH-regulating effects [109]. Moreover, they are a superior alternative to synthetic protective agents in enriched foods and limit the formation of undesirable processing-derived compounds through free-radical scavenging and metal-chelating activity [110]. The reduction in oxidative reactions may suppress the formation of Maillard intermediates, mitigating their undesirable effects [111]. The hydroxyl groups and aromatic rings of phenols in rosemary, turmeric, and bay leaf play a crucial role in inhibiting the formation of hazardous compounds such as malondialdehyde, glyoxal, methylglyoxal, heterocyclic aromatic amines, acrylamide, and 5-hydroxymethylfurfural in processed meat products [112]. Moreover, the mixed application of grape seed extract and fish gelatin in fish products resulted in an inhibition of the growth of spoilage bacteria in the microbiota, thereby reducing the accumulation of biogenic amines (putrescine and cadaverine), and it helped maintain the moisture state of fish by acting as a gas/water barrier and suppressing the transformation from immobilized water captured in the myofibrillar protein network [113]. These activities are strongly dependent on the phytochemical composition of the herb or spice, its concentration, the meat matrix, cooking temperature and duration, and the specific hazardous compound considered. As seen in Table 5 [11,71,112,114,115,116,117,118,119,120,121,122,123,124,125], the use of herbal nutraceuticals contributes various functional characteristics to meat products. The incorporation of herbs, spices, plant extracts, and essential oils in meat products primarily reduces harmful compounds and enhances antioxidant properties due to their rich phenolic content.

## 4. Novel Delivery Technologies for Nutraceuticals

Including nutraceuticals in the food matrix presents significant technological challenges. Various bioactive compounds are unstable and easily degraded during production and storage. Encapsulation techniques such as emulsification, spray drying, lyophilization, extrusion, and electrospraying are widely used to protect the stability of nutraceuticals during food processing [126]. Novel delivery systems that preserve the integrity and efficacy of nutraceuticals throughout food processing and the human digestive system have been developed. The nanoencapsulation technique increases the absorption and health effects of bioactive compounds, and stimuli-responsive systems enable accurate nutrient release in the gastrointestinal tract [127]. In the delivery of phytochemicals in food matrices, electrospun nanofibers provide high loading and sustained nutrient release, while nanoemulsions offer ameliorated solubility, swift uptake, and shelf life of compounds [128]. Additionally, a class of nanoparticles, classified as two-dimensional (2D) materials such as MXenes, layered double hydroxides, metal–organic frameworks, transition-metal dichalcogenides, and boron nitride nanosheets, is characterized by their atomic thickness and large lateral dimensions and can be used to effectively encapsulate bioactives, thereby improving their solubility, chemical stability, and the controlled or stimuli-responsive release of polyphenols, flavonoids, vitamins, peptides, and probiotics [129]. In encapsulation technology, bioactive peptides, especially those rich in proline, exhibit digestion resistance and self-assembling characteristics, successfully encapsulate active molecules, and enhance the flavor of food products [130].

Despite their potential advantages, nanoscale delivery systems are particularly effective in enhancing the dispersion, stability, and functional performance of hydrophobic bioactive compounds, whereas controlled-release systems offer extended protection but often exhibit reduced predictability when transferred from in vitro systems to real meat matrices [131].

## 5. Stability of Nutraceuticals in Processing, Storage and Digestion

Chemical stability and effectiveness of nutraceuticals are affected by abiotic (temperature, light, oxygen, pH, water activity, nanoformulation, extraction process, and urbanization) and biotic (microorganisms, mycorrhizal symbiosis, genetic modification, climate change, and biodiversity loss) environmental factors during processing and storage [132]. Changes in these factors accelerate the degradation of bioactive compounds, particularly exposure to light and high water activity. Grinding promotes the release of bioactive substances [133], and fermentation drives the enhancement of bioactives [134] during the production process. The fatty acid profile of oils in meat products is not markedly affected by processing, such as heat treatment, curing, drying, or smoking. Conversely, replacing animal fat with oleogels or plant-based oils increases the product’s unsaturated fatty acid content and nutritional quality [14,79,85,135]. Furthermore, many studies indicated that the functional properties of meat products acquired from nutraceuticals are maintained during storage [69,71,79,86,98]. In the meat matrix, polyphenols interact with proteins by covalent or non-covalent bonds and construct protein–polyphenol complexes that prevent the lipid oxidation of the product, reduce protein oxidation, and promote protein solubility. These reactions that enhance functional attributes of proteins depend on the proteins’ hydrophobic/hydrophilic character and the reaction sites accessible for polyphenols [136]. Plant polyphenols and extracts such as tannic acid, catechin, carvenol, coffee extracts, granatum extracts, and rape bee pollen have a reducing effect on lipid oxidation levels and contribute to the color of meat, meat products, and certain fish [137]. The addition of chlorogenic acid, ferulic acid, gallic acid, and tannic acid polyphenols into sausage formulation promoted gastric proteolysis but suppressed intestinal proteolysis at high levels. In contrast, chlorogenic acid and gallic acid inhibited gastric proteolysis but increased intestinal digestion at low levels [68]. Processing-induced structural changes, particularly heat-induced protein denaturation, aggregation, and gel formation, may further influence the disintegration of the digestive matrix and the gastrointestinal release of nutraceuticals. Overall, the available literature indicates that the stability of nutraceuticals incorporated into processed meat products is highly dependent on the physicochemical properties of the bioactive compound, its form of incorporation, interactions with the meat matrix, processing conditions, packaging, and storage period. Although encapsulation, lipid structuring, or favorable matrix interactions may enhance the retention of certain compounds, other nutraceuticals may remain susceptible to oxidation, thermal degradation, and reduction in biological activity or microbial viability. Therefore, stability should be regarded as a compound-, formulation-, matrix-, processing-, and storage-dependent property rather than as an inherent consequence of incorporation into processed meat products.

## 6. Bioaccessibility and Bioavailability of Nutraceuticals in Meat Products

The term bioavailability involves absorption, delivery, metabolism, and elimination of bioactive compounds and nutrients [138]. Bioaccessibility refers to the fraction of an ingested biocomponent that becomes accessible for gastrointestinal absorption and is determined in an in vitro digestion model [139]. The processed meat matrix can influence bioavailability and bioaccessibility through multiple mechanisms. Interactions of biocomponents with proteins, lipids, minerals, and other matrix constituents may alter their release during digestion, whereas thermal processing, protein aggregation, lipid oxidation, encapsulation, and the physical form of incorporation may further modify digestive accessibility. Lipid-rich phases may facilitate the solubilization and micellar incorporation of lipophilic compounds, while strong interactions between polyphenols and proteins may either protect bioactive compounds during processing and digestion or restrict their release from the matrix. Therefore, the amount of a nutraceutical retained in the product after processing should be distinguished from the amount released during digestion and, subsequently, from the amount absorbed in vivo. The reported outcomes of in vitro meat matrix studies are summarized below. Bako et al. [140] investigated the bioavailability of encapsulated rosmarinic acid and β-carotene in beef sausages. They determined that encapsulation and addition into the meat matrix can improve the bioavailability of these bioactive dietary components, potentially leading to the production of health-oriented meat products. Bolger et al. [141] evaluated the bioaccessibility of alpha-linolenic acid (ALA) from flaxseed oil added directly, emulsified, and encapsulated (three types) in chicken sausages. Results indicated that the bioaccessibility of ALA ranged from 19.2% to 33.2%, with the highest value observed in freeze-dried encapsulated flaxseed oil samples. Azizi et al. [142] revealed that the incorporation of carrot puree was ineffective for the bioaccessibility of protein, fiber, and iron, while moringa leaf puree stimulated the bioaccessibility of iron in beef sausage. Furthermore, increased bioaccessibility of fatty acids at digestion and improved protein hydrolysis during mid-duodenal digestion relative to control were identified in in vitro tests of salami produced with grapeseed, green tea, and olive mixed plant extracts [143]. Incorporation of *Spondias bahiensis* bagasse extract into goat meatballs enhanced the bioaccessibility of catechin, gallic acid, epicatechin gallate, and procyanidins B1 and B2 compared with the control [72]. Calcium lactate and calcium citrate malate-enriched dry fermented sausages exhibited close to 10% (good) calcium bioavailability in an in vitro assay, with high sensory quality [144].

Studies conducted in humans provide data for evaluating the systemic bioavailability of nutraceutical-enriched meat products. However, the scarcity of the literature on human intervention is a major limitation. The bioavailability and tolerability of eicosapentaenoic acid (EPA)- and docosahexaenoic acid (DHA)-enriched sausages were investigated, and an increased omega-3 index among enriched sausage consumers compared with placebo consumers was reported [145]. Based on the summarized literature, the meat matrix may be a convenient medium for enhancing the bioavailability of nutraceuticals due to the affinity of meat proteins for these compounds.

## 7. Health Benefits of Nutraceutical-Enriched Meat Products

Nutraceutical intake is associated with promising outcomes for disease prevention through multiple protective mechanisms in human health. Nutraceuticals are effective in treating cardiovascular diseases, Alzheimer’s disease, cancer, diabetes, obesity, osteoarthritis, eye disorders, and stress management [146]. Clinical studies supply the evidence for assessing whether incorporating nutraceuticals into processed meat products achieves measurable physiological effects (Table 6) [145,147,148,149,150,151,152]. However, compared with the extensive literature addressing technofunctional characteristics, sensory acceptability, and meat product reformulation, the number of controlled human studies on the consumption of these innovative foods remains limited. Available research reports have evaluated diverse formulations, with outcomes mainly related to postprandial metabolism, blood lipid levels, inflammatory markers, antioxidant biomarkers, gut microbial community, and other cardiometabolic endpoints. Nevertheless, considerable heterogeneity among study designs, participant characteristics, consumption periods and amounts, and monitored values currently limits direct comparisons among studies. It precludes broad conclusions regarding the health benefits of nutraceutical-enriched processed meats.

In addition to human studies, animal feeding experiments have also been conducted on the consumption of nutraceutical-containing meat products. Dietary consumption of carob fruit extract-enriched meat per day for 8 weeks in rats resulted in a lower *Enterobacteriaceae* abundance, higher levels of SCFA production, and better colonic barrier integrity than control animals [153]. Another study on rats indicated that carotenoids, omega-3 fatty acids, catechins, carnosic acid, propyl propane thiosulfonate, a postbiotic from *Lactiplantibacillus plantarum*, α-tocopherol, and ascorbyl palmitate incorporated into processed meat product consumption during 20 weeks per day achieved potent antitumor effects on colorectal cancer and modulated the gut microbiome [154]. The immunomodulating effects of fermented beef meat feeding, including probiotic *Lactobacillus plantarum*, *Lactobacillus paracasei*, *Pediococcus acidilactici*, and *Staphylococcus carnosus* strains, were investigated in sensitized rats. This experiment showed that fermented meat consumption has an effect on reducing the risk of food allergies, with a 43.5% decrease in the intensity of the anaphylactic reaction, lower concentrations of specific antibodies (Immunoglobulin G) to ovalbumin in the blood serum, and no morphofunctional changes in the mesenteric lymph node structures on histological examination compared to the control [155]. Although clinical research on certain nutraceuticals in meat products is scarce, their inclusion offers many health benefits, such as reducing the risk of cardiovascular diseases, food allergies, obesity, and inflammatory markers, as well as supporting the intestinal system.

## 8. Future Perspectives

The development of next-generation meat products will increasingly rely on characterizing the behavior of nutraceuticals within the meat matrix. For future applications, it is necessary to focus on detailed insights into molecular interactions among meat proteins, lipids, carbohydrates, and incorporated bioactive compounds, using advanced analytical techniques, including metabolomics, proteomics, and molecular imaging. Additionally, innovative delivery systems should be optimized specifically for processed meat applications. The health effects of nutraceutical-enriched meat product consumption require more robust biological evidence from placebo-controlled randomized human clinical trials. Future research should focus on measurable health outcomes of consumption such as cardiometabolic risk factors, irritable bowel syndrome, inflammation, oxidative stress, and immune function. Sustainability and clean-label reformulation strategies should be yet another issue for functional meat product development. Personalized nutrition strategies and consumer-oriented reformulation for people with specific physiological requirements and health conditions should be yet another focus for functional meat product development.

## 9. Conclusions

The meat matrix differs markedly from other food matrices in its high protein and fat content, product variety, and wide range of production conditions. Incorporating nutraceutical compounds into the meat matrix substantially improves the technological quality, oxidative stability, microbial safety, nutritional profile, and sensory characteristics of processed meat products. Available in vitro studies indicate that meat–matrix interactions and delivery strategies can influence the digestive release and bioaccessibility of incorporated compounds; however, evidence regarding systemic bioavailability remains substantially more limited, particularly in humans. Further animal and human studies on the consumption of nutraceutical-enriched meat products are therefore required to determine whether improved processing stability or digestive release translates into high gastrointestinal absorption and clinically significant physiological effects. Overall, nutraceutical-enriched processed meats represent an innovative approach for improving both product functionality and nutritional value, supporting the development of healthier, clean-label, and evidence-based functional meat products.

## Figures and Tables

**Table 1:** Recent applications of dietary fiber in meat products.

Bioactive/Functional Ingredient	Product	Dosage (Method)	Reported Effects/Outcomes	Reference
Plantago husk	Beef salami	3% (direct powder)	Technological: improved technological properties; enhanced emulsion stability and cooking yield	[12]
Amaranth flour	Chicken snack sticks	15% (direct powder)	Technological: improved flavor and sensory acceptance	[45]
Coconut flour	Beef sausage	10% (direct powder)	Technological: improved sensory quality Nutritional/functional: improved dietary fiber content Preservation: better oxidative stability	[46]
Lemon fiber	Beef sucuk	1–2% (direct powder)	Technological: decrease cooking loss; stabilized texture, pH, and color properties Preservation: stabilized thiobarbituric acid reactive substances (TBARSs)	[47]
Wheat starch, wheat gluten	Fish nugget	14% (direct powder)	Technological: composite gel network formation, alterations in crust characteristics; ultimately inhibits oil penetration	[48]
Hazelnut skin powder	Beef burger	10% (direct powder)	Technological: decreased L*, a*, b* values, improved texture and organoleptic quality Nutritional/functional: increased oleic acid and linoleic acid content	[49]
Date seed powder, quinoa flour	Fish, chicken nuggets	6% (direct powder)	Technological: improved water-holding capacity and cooking yield; lower oil absorption; better sensory attributes Nutritional/functional: increased dietary fiber content	[50]
Spent coffee grounds	Beef patties	10% (direct powder)	Technological: strong protein binding interactions; improved color properties; high consumer acceptability Preservation: decreased TBARS	[51]
Inulin	Buffalo sausage	6% (direct powder)	Technological: increased fiber; decreased fat; improved sensory characteristics	[52]
Psyllium husk	Chicken sausage	2% (direct powder)	Technological: stability of technological and sensory properties Nutritional/functional: nutritional enrichment	[13]
Rice bran	Chicken sausage	10–15% (direct powder)	Technological: improved sensory properties; maintained cooking loss	[53]
Orange fiber, wheat fiber, bamboo fiber, carrot fiber	Chicken sausage	1% (direct powder)	Technological: maintained pH and color properties (wheat fiber); increased pH and darkest color (bamboo fiber and carrot fiber) Preservation: decreased TBARS (orange fiber)	[54]
Tomato peel flour	Beef burger	4% (direct powder)	Technological: increased water-holding capacity; hard texture; less gumminess; darker, reddish, yellowish color Nutritional/functional: decreased moisture and fat Preservation: delayed lipid oxidation during cooking	[55]
Apple, inulin, pea, oat fibers	Meat loaf	6% (direct powder)	Technological: decreased cooking loss; improved water retention capacity during storage (apple, inulin, pea, oat fibers); improved texture, water retention, and sensory properties (pea and oat fibers) Preservation: decreased TBARS (inulin and oat fibers)	[56]
Carrot, ginger	Chicken nugget	13% (direct powder)	Technological: increased color and sensory properties Preservation: decreased TBARS and microbial growth	[57]
Avocado pulp, peel powder	Fish meatball	3% (direct powder)	Nutritional/functional: increased polyunsaturated fatty acids (PUFAs), monounsaturated fatty acids (MUFAs) and omega-3 fatty acids Preservation: reduced bacterial counts; decreased total volatile basic nitrogen	[58]

**Table 2:** Applications of polyphenols and polyphenol-rich ingredients in meat products.

Bioactive/Functional Ingredient	Product	Dosage (Method)	Reported Effects/Outcomes	Reference
Chlorogenic acid, ferulic acid, gallic acid, tannic acid	Pork sausage	0.04% (direct solution)	Technological: reduced particle size Nutritional/functional: increased gastric digestion and viscoelasticity; reduced intestinal digestion	[68]
*Psidium guajava*, *Ecklonia cava*, *Paeonia japonica* extracts	Pork sausage	0.5% (directly lyophilized)	Preservation: reduced TBARS during storage; inhibited pathogen growth Technological: high overall acceptance	[69]
Ferulic acid	Dried meat	0.1% (directly dissolved in brine)	Preservation: decreased lipid oxidation; reduced total aerobic mesophile counts Nutritional/functional: increased antioxidant activity	[70]
Olive polyphenols	Pork sausage	0.2% (directly dissolved in water)	Preservation: decreased malondialdehyde during storage Technological: high yellowness; shear force and cooking loss stability	[71]
*Spondias bahiensis* bagasse	Goat meatball	7% (direct powder)	Nutritional/functional: high antioxidant activity; increased bioaccessibility of catechin, epicatechin gallate, procyanidins B1, gallic acid, and procyanidin B2	[72]
*Prunus mume* polyphenol	Pork sausage	0.9 g/kg (directly freeze-dried)	Preservation: reduced TBARS; inhibited N-nitrosamine accumulation Technological: improved texture; color stability	[73]
Coffee extract (chlorogenic acid)	Pork sausage	0.32% (direct powder)	Preservation: stabilized lipid oxidation Technological: maintained sensory attributes	[74]
Flavonoids	Heated meat	35 mg L^−1^ (directly dissolved in ethanol)	Preservation: inhibited polycyclic aromatic hydrocarbon (PAH) formation Nutritional/functional: strong antioxidant properties and free-radical scavenging abilities	[75]
Catechins	Meat batter	1500 mg/kg (directly dissolved in water)	Preservation: inhibited TBARS Technological: promoted drip loss; excessive expansion and aggregation between myofibrillar proteins; reduced interfacial proteins	[76]
Tannic acid—whey protein	Pork sausage	1.5% (directly dissolved in water)	Preservation: decreased TBARS during storage Technological: stabilized lipid and physicochemical properties	[77]
Ferulic acid	Horse meat sausage	0.72% (encapsulated)	Preservation: reduced tyramine accumulation; antibacterial properties; decreased *Enterobacteriaceae* level	[78]

**Table 3:** Incorporation of plant-based oils and structured lipid systems into meat products.

Bioactive/Functional Ingredient	Product	Dosage (Form)	Reported Effects/Outcomes	Reference
Carnauba wax	Beef burger	Replaced at 53% (oleogel)	Nutritional/functional: Increased PUFAs Technological: reduced cooking shrinkage; improved fat and water retention	[14]
Soybean oil	Pork sausage	7.5% (emulsified gel)	Nutritional/functional: Increased PUFAs and MUFAs; lower cholesterol content Technological: decreased released water and fat; increased hardness and overall acceptability	[15]
Pumpkin seed oil	Buffalo sausage	2% (liquid)	Nutritional/functional: improved PUFA profile Preservation: decreased TBARS; protective effect against spoilage bacteria	[52]
Avocado, sea buckthorn, macadamia oils	Buffalo sucuk	12.5% (liquid)	Nutritional/functional: improved PUFA profile and health-related lipid indices Technological: stability of L* and a* values during storage	[79]
Pumpkin seed oil	Buffalo salami	0.5% (liquid)	Technological: optimal sensory characteristics Preservation: microbiological safety; decreased lipid oxidation	[81]
Canola oil	Beef burger	7.5% (bigel)	Technological: improved thermal stability and water-holding capacity; reduced hardness, chewiness, and gumminess	[82]
Sunflower oil	Pork patties	7% (oleogel)	Nutritional/functional: improved fatty acid profile Technological: contribution to meaty aroma, saltiness, and yellow-brown color	[83]
Pine nut oil	Horse meat, beef, chicken mixed sausage	10% (emulsion gel)	Nutritional/functional: PUFA/SFA ratio of 1.00; reduced atherogenic and thrombogenic indices Technological: improved emulsion stability, cooking yield, and water retention	[84]
Linseed, walnut, algal oils	Chicken sausage	25% (emulsion gel)	Nutritional/functional: improved atherogenic and thrombogenic indices; increased hypocholesterolemic/hypercholesterolemic ratio Preservation: oxidative stability Technological: sensory acceptability	[85]
Sacha inchi oil	Pork chorizo	3–6% (encapsulated)	Nutritional/functional: improved PUFA profile; Preservation: stability of oxidation during storage Technological: stability of hardness during storage	[86]
Chia, hemp oils	Beef burger	5–10% (emulsion gel)	Nutritional/functional: improved PUFA profile Technological: improved cooking characteristics; consumer acceptance	[87]

**Table 4:** Incorporation of probiotics and postbiotics into meat products.

Bioactive/Functional Ingredient	Product	Reported Effects/Outcomes	Reference
*Lactiplantibacillus plantarum*, *Staphylococcus xylosus*	Pork sausage	Preservation: inhibited lipid oxidation; preserved volatile compounds Nutritional/functional: antioxidant effect	[94]
*Bacillus amyloliquefaciens* MS15a	Pork sausage	Preservation: improved microbial safety and product quality Nutritional/functional: increased organic acid levels	[95]
*Lacticaseibacillus rhamnosus* E26, *Lactiplantibacillus plantarum* M5.1	Buffalo salami	Nutritional/functional: reduced cholesterol; increased PUFAs Preservation: improved microbiological safety Technological: improved aroma and texture	[96]
*Pediococcus acidilactici* 0110<TAT-1	Fish sausage	Preservation: accelerated pathogen reduction Technological: increased pH	[97]
Postbiotics (derived from *Lactobacillus bulgaricus*)	Beef sausage	Preservation: stability of total volatile base nitrogen during storage; inhibition of mesophilic and psychrophilic bacteria, mold and yeast growth Technological: stability of pH, moisture, and fat	[98]
Postbiotics (derived from *Lactobacillus acidophilus*)	Pork sausage	Nutritional/functional: increased protein and organic acid contents Technological: improved cohesiveness, springiness; pH, color stability	[99]
*Pediococcus pentosaceus* 128b, *Latilactobacillus sakei*, *Lactiplantibacillus plantarum*, *Staphylococcus carnosus*	Beef sucuk	Nutritional/functional: increased phenols, furans, acids, terpenes, aromatic hydrocarbons, ketones, nitrogenous compounds, esters, and aliphatic hydrocarbons Preservation: inhibited *Micrococcus*/*Staphylococcus* levels Technological: lower pH	[100]
*Lactiplantibacillus plantarum*, *Enterococcus faecium*, *Enterococcus faecalis*	Beef sucuk	Preservation: reduced TBARS Technological: increased L* and a* values during storage; improved textural values, taste, and general acceptability	[101]
*Lacticaseibacillus rhamnosus* LR32 200B, *Lactiplantibacillus plantarum* LP115 400B, *Bifidobacterium lactis* BB12	Beef salami	Nutritional/functional: increased moisture and fat content Preservation: 6 log CFU/g lactic acid bacteria count at 60 days of storage Technological: decreased pH; improved sensory quality	[102]
*Lactobacillus sakei,* *Lactiplantibacillus plantarum*, *Pediococcus pentosaceus*	Pork sausage	Preservation: thiobarbituric acid reactive substances and sodium nitrite levels Technological: improved color and texture; high sensory scores; reduced pH	[103]
*Lactiplantibacillus plantarum* BFL	Pork sausage	Preservation: decreased residual nitrites; decreased *Enterobacteriaceae* and *Staphylococcus* counts Technological: maintained sensory attributes	[104]
*Lacticaseibacillus casei* SJRP38, *Lc. casei* SJRP146, *Lc. paracasei* BGP1, *Lc. rhamnosus* LGG	Pork sausage	Preservation: decreased lipid oxidation levels during storage Technological: reduced water activity, pH; improved texture	[105]

**Table 5:** Incorporation of herbs, spices, plant extracts, and essential oils into meat products.

Bioactive/Functional Ingredient	Product	Dosage (Form)	Reported Effects/Outcomes	Reference
Curcumin	Chicken nugget	0.5% (oleogel coating)	Nutritional/functional: suppressed advanced glycation end products and 5-hydroxymethylfurfural formation during frying Technological: improved texture and color; preserved sensory quality of fried chicken	[11]
Rosemary, savory, camellia, thyme, lemon balm, turmeric mix	Pork sausage	0.2% (emulsified)	Preservation: decreased malondialdehyde during storage Technological: greater pH value; high yellowness; stability under shear force and cooking loss	[71]
Rosemary, turmeric, bay leaf	Beef patties	0.5% (dissolved in ethanol)	Nutritional/functional: decreased hazardous compounds; reduced amino acid retention Technological: reduced cooking loss and water loss; improved texture	[112]
Artichoke powder	Beef sausage	10% (powder)	Preservation: enhanced oxidative and microbial stability; decreased thiobarbituric acid and total volatile basic nitrogen values Technological: sensory acceptance	[114]
Eugenol	Horse meat sausage	3.1% (nanoemulsion)	Preservation: reduced histamine accumulation; suppressed inoculated pathogens; stability of the microbial community	[115]
Mulberry, olive, guava leave extracts	Fish meatball	1.5% (dissolved in ethanol)	Nutritional/functional: improved antioxidant properties; increased protein value Preservation: extended shelf life	[116]
Scent leaves, ginger rhizome	Smoked African catfish	2% (powder, solution)	Preservation: reduced TBARS and peroxide value; decreased total trimethylamine nitrogen level Technological: improved sensory quality	[117]
Barberry extract	Beef sausage	400 mg/kg (dissolved in ethanol)	Preservation: inhibited lipid–protein oxidation; reduced peroxide values, TBARS, and carbonyls; reduced nitrosamine and 3-NT levels during cooking	[118]
Black garlic	Sucuk	3% (powder)	Preservation: decline in bacteria counts; lower residual nitrite level; effect on N-nitrosopiperidine formation after 3 min of cooking	[119]
Sage, ginger extracts	Beef, pork mixed salami	2% (dissolved in ethanol)	Nutritional/functional: strongest antioxidant activity; increased phenolic content Preservation: reduced oxidation	[120]
Rosemary	Sucuk	250 mg/kg (oleoresin)	Preservation: increased TBARS during storage; stability against bacterial growth	[121]
Caraway seeds, caraway essential oil, Juniper berries	Pork sausage	0.1% (solution)	Preservation: increased peroxides Technological: improved organoleptic properties; extended shelf life	[122]
Celery	Beef sausage	150 mg/kg (powder)	Preservation: increased N-nitrosopiperidine; stability of residual nitrite and volatile compound profiles Technological: increased pH; decreased water activity	[123]
*Malva parviflora* leaves	Beef sausage	1.5% (powder)	Nutritional/functional: improved protein and dry matter levels Technological: decreased pH during storage; slight decrease in color and taste properties	[124]
Chia, black cumin seeds	Pork sausage	2% (powder)	Technological: improved volatile compounds, particularly terpenes; high herbal odor and flavor	[125]

**Table 6:** Summary of human intervention studies evaluating nutraceutical- or functional ingredient-enriched processed meat products.

+	Participants	Design	Main Findings	Reference
Beef hamburger meatball enriched with 7.5% psyllium	25 healthy adults (19–35 years, BMI 18.5–25 kg/m^2^); randomized, placebo-controlled, crossover	2 h after 160 g meatball consumption, postprandial blood samples	Decreased the postprandial rise in triglyceride and very-low-density lipoprotein levels; decreased glucose levels; reduced hunger; increased satiety	[147]
Fish sausage enhanced with DHA 1010 mg and EPA 240 mg	20 normolipidemic adults (20–60 years); randomized, double-blind, placebo-controlled	Consumed 50 g per morning for 8 weeks; blood samples	Decreased serum total SFA; suppressed serum triglyceride levels; decreased serum n-6 polyunsaturated fatty acids	[148]
Fermented pork salami with *Lactobacillus rhamnosus* HN001 and citrus fiber	24 healthy adults (20–30 years, BMI 18.5–25 kg/m^2^); randomized, double-blind, placebo-controlled	Consumed 30 g per day for 4 weeks; anthropometric measurements, blood and fecal samples	Decreased inflammatory markers such as C-reactive protein (CRP) and tumor necrosis factor-alpha (TNFα); improvement in antioxidant plasmatic markers; increased fecal butyrate production; stability in the gut microbiota	[149]
Beef steaks and frankfurter sausages prepared with 20% walnuts	25 overweight volunteers (45–70 years, BMI 25–34 kg/m^2^); placebo-controlled	Consumed 150 g steak (4 per week) and 80 g frankfurter (1 per week) for 5 weeks; blood samples	Decreased total cholesterol and low-density lipoprotein cholesterol; increased α-tocopherol; decrease in body weight	[150]
Chicken meat enriched with 500 mg of n-3 PUFAs	39 healthy young individuals; blinded, placebo-controlled	Consumed 500 g per day for 3 weeks; blood samples	Reduced high-sensitivity CRP; increased antioxidant defense marker levels; increased glutathione peroxidase (GPx) and superoxide dismutase (SOD) enzyme activities	[151]
Burger enriched inulin-based hydrogel	39 normolipidemic adults (18–58 years); placebo-controlled	Consumed 100 g 3 times per week for 8 weeks; blood and fecal samples, breath tests	Reduced triglycerides; enrichment of *Akkermansia* and SCFAs	[152]
Sausages enriched with 250 mg EPA and DHA	44 low omega-3 index adults (18–34 years); randomized, double-blind, placebo-controlled	Consumed 80 g per day for 8 weeks; blood samples	Increased omega-3 index; stability in ALA levels; increased docosapentaenoic acid levels	[145]

## Data Availability

No new data were created or analyzed in this study. Data sharing is not applicable to this article.

## Abbreviations

The following abbreviations are used in this manuscript:

ALA	Alpha-linolenic acid
CRP	C-reactive protein
DHA	Docosahexaenoic acid
EPA	Eicosapentaenoic acid
GPx	Glutathione peroxidase
*Lc*	*Lacticaseibacillus*
MUFAs	Monounsaturated fatty acids
PAH	Polycyclic aromatic hydrocarbons
PUFAs	Polyunsaturated fatty acids
SCFAs	Short-chain fatty acids
SFAs	Saturated fatty acids
SOD	Superoxide dismutase
TBARSs	Thiobarbituric acid reactive substances
TNFα	Tumor necrosis factor-alpha
TVBN	Total volatile basic nitrogen
